# Salivary metabolomics in patients with oral lichen planus: a preliminary study based on NMR spectroscopy

**DOI:** 10.1007/s00784-023-05389-1

**Published:** 2024-01-18

**Authors:** Bina Kashyap, Eelis Hyvärinen, Igor Laitinen, Arja M. Kullaa

**Affiliations:** 1 Department of Oral Diagnostic Sciences, Faculty of Health Sciences, Institute of Dentistry, University of Eastern Finland, Kuopio Campus, PO Box 1627, 70211 Kuopio, Finland; 2 Oral, Private Clinic, Lahti, Finland

**Keywords:** Saliva, Salivary metabolites, Oral lichen planus, Tyrosine, Methylamine, Periodontitis

## Abstract

**Objectives:**

The present preliminary study aimed to investigate the salivary metabolic profile in patients with asymptomatic oral lichen planus (OLP) using nuclear magnetic resonance (NMR) spectroscopy.

**Material and methods:**

Stimulated whole mouth saliva (SWMS) samples were collected from 15 reticular OLP female patients and 15 from age- and sex-matched controls (HCs). A total of 23 metabolites were identified and quantified. Mann–Whitney’s *U* test was used to compare the determined concentration salivary metabolite concentrations between OLP patients and the healthy controls.

**Results:**

The concentration of acetate, methylamine, and pyruvate was elevated, whereas the concentration of tyrosine was decreased in the saliva of OLP patients compared with HCs. To identify a combination of metabolites, multivariate discrimination function analysis (DFA) was conducted. DFA analysis have shown that the most powerful discrimination between the groups was achieved when methylamine and tyrosine were considered as combined biomarkers.

**Conclusions:**

Salivary tyrosine was of particular interest and a promising finding for the screening of OLP and its progression. Further longitudinal studies are required to establish it as a reliable salivary biomarker in OLP.

**Clinical relevance:**

The salivary metabolic profiling can describe the pathologic characteristics of OLP on non-invasive saliva samples and NMR analysis. Salivary metabolites provide details to considered early detectors and to impact oral health of OLP patients.

## Introduction

Oral lichen planus (OLP) is a chronic immune-mediated inflammatory disease affecting oral mucosa with characteristic relapses and remissions [1]. OLP is a potentially precancerous condition, defined as a generalized state associated with a significantly increased risk of oral cancer [2]. Risk factors for malignant transformation include ulceration, location on the tongue, and female sex [2]. The extent of involvement with OLP is variable. There are six clinical variants of OLP: reticular, papular, plaque, atrophic, erosive (ulceration), and bullous. The pathogenesis of OLP is controversial, and several theories such as T-cell mediated chronic inflammatory reaction and antigen-specific and non-specific mechanisms have been put forward [3]. Genetic factors and other potential factors like stress, trauma, oral habits, and dental procedures can contribute to OLP pathogenesis [4, 5]. The diagnosis of OLP was performed clinically and with histological examinations but considering its malignant potential further progress in understanding the basis of carcinogenesis is emphasized. There are several genes and proteins reported as biomarkers for clinical diagnosis but with insufficient diagnostic sensitivity and specificity [6, 7]. Asymptomatic lesions do not require treatment; for symptomatic patients, topical corticosteroids (e.g., triamcinolone) are typically used. Topical calcineurin inhibitors, intralesional corticosteroid injections, and systemic agents (e.g., prednisone) are reserved [8].

The changes in the metabolic profile of the biofluids in a variety of physiological and pathological processes signal the presence of a disease state [9]. Saliva is an important biofluid that contains a large collection of proteins and peptides. The proteins and metabolic end products that are expressed in saliva are known to alter greatly in several diseases [10]. Salivary proteomics on OLP has shown reduced “palate, lung and nasal epithelium carcinoma-associated protein” (PLUNC) as a novel biomarker [11]. In another study [12], a positive correlation of salivary proteomes with inflammatory cytokines and OLP was reported. There are several studies where the salivary biomarkers are identified with the clinical relevance to impact the oral and general quality of life of OLP patients (Table 1).

**Table 1:** Salivary biomarkers identified as diagnostic, prognostic and progression marker in oral lichen planus (OLP)

Oral diseases	Saliva	Elevated salivary biomarker	Clinical relevance	Reference
OLP	WUS	Cortisol	Diagnostic biomarker	[13–17]
OLP	WUS	Nitric oxide (NO)	Diagnostic and prognostic potential	[18, 19]
OLP	WUS	Reactive oxygen species, ROS	Oxidative stress	[18, 20]
OLP	WUS	C-reactive protein, CRP	Inflammation marker	[18, 21, 22]
OLP	WUS	TNF-α	Diagnostic marker	[19, 23, 24]
OLP	WUS	IL1, IL4, IL6, IL8	Prognostic marker	[25–33]
OLP	USWS	Trimethylamine *N*-oxide, putrescine, creatinine, 5-aminovalerate, pipecolate, *N*-acetylputrescine, gamma-butyrobetaine, indole-3-acetate, *N*_1_-acetylspermine, 2′-deoxyinosine, ethanolamine phosphate, *N*-acetylglucosamine	Disease progression	[34]
OLP	WUS	Mathematical model	Diagnostic method	[35]
OLP	USWS	6 amino acid metabolites, 2 carnitines, 2 lipid metabolites and 9 other metabolites	Early diagnosis of OLP	[36]

*WUS* whole unstimulated saliva, *USWS* unstimulated whole saliva, *TNFα* tumor necrosis factor alpha, *IL* interleukin

Various analytic techniques such as high-resolution nuclear magnetic resonance (^1^H-NMR) spectroscopy, high-performance liquid chromatography-mass spectroscopy (HPLC–MS), and gas chromatography (GC–MS) are applied in salivary metabolic profiles for detecting changes [37]. There are very few previous MS studies that have shown salivary metabolite’s potential to discriminate OLP from oral cancer and controls. Salivary metabolic studies have provided a comprehensive quantitative analysis of all the metabolites, but most of the studies focused on oral cancer for the clinical benefit of early detection. One study showed indole-3-acetate and ethanolamine phosphate alterations in OLP whereas in another study, 19 metabolites were identified in salivary samples of OLP [34, 36]. However, the use of ^1^H-NMR spectroscopy in salivary studies is very limited when compared to other analytical methods.

Determining saliva metabolites in patients with OLP using NMR can be highly advantageous in early detection, disease prognosis, and selecting the most appropriate treatment modalities. Hence, we aimed to study salivary metabolic changes associated with reticular OLP using NMR spectroscopy. The hypothesis was that there would be differences in the quantitative measurement of the salivary metabolic profile of OLP, and these differences could provide good diagnostic potential for early detection.

## Materials and methods

### Subjects

The present study was conducted according to the guidelines of the Declaration of Helsinki and was approved by the Oulu University Hospital Ethical Committee (EETTMK; 36/2012) and the Research Ethics Committee of the Northern Savo Hospital District (754/2018; 21.4.2020). The present study fulfilled the World Medical Association Declaration (Helsinki, Finland, 1964). Before participating in this study, all participants were fully informed, and signed written consent was obtained.

**Fig. 1:**
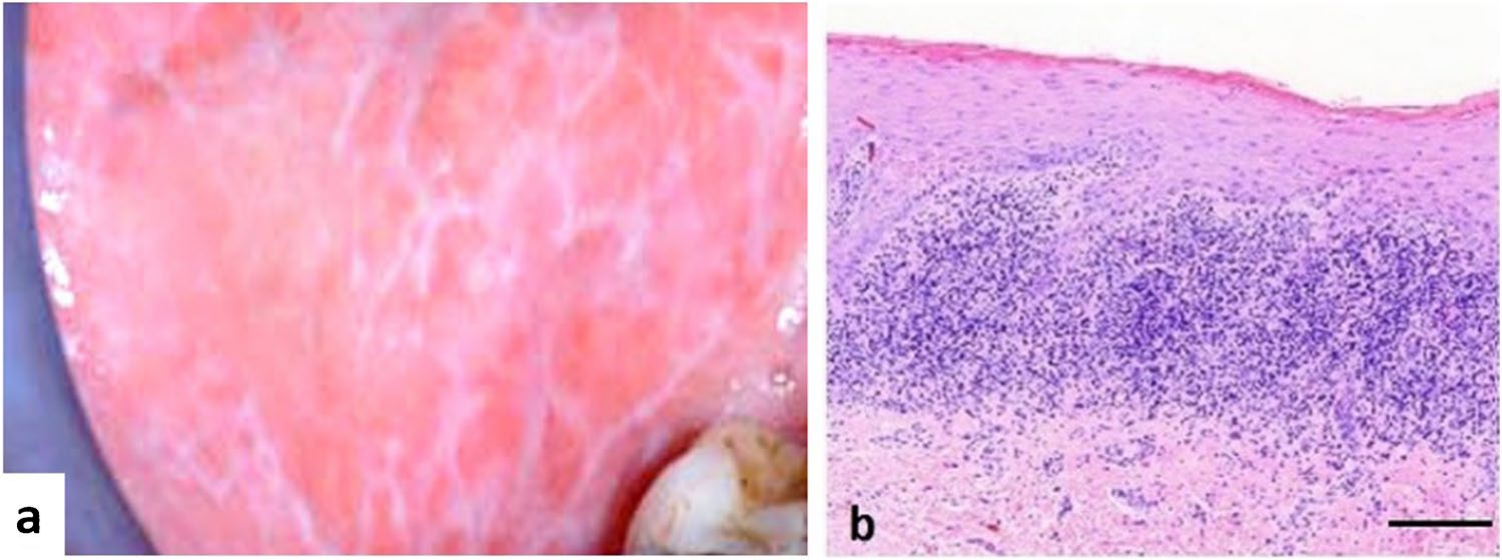
Oral lichen planus showing **a** typical clinical appearance of reticular form showing white lines (Wickham’s striae) located symmetrically on the buccal mucosa; **b** histological specimen shows mild subepithelial lymphocytic infiltration (Bar = 100 µm)

The material of this study consists of 15 patients with reticular OLP from 45 consecutive patients with OLP. The clinical appearance of asymptomatic OLP was typical reticular OLP which were diagnosed clinically by an author (AMK) and verified histologically (Fig. 1). Patients with erosive, bullous, and atrophic OLP or having medication for oral symptoms have been excluded from the study.

All patients with a history of any other medical diseases and smoking habits were excluded from this study. As healthy controls (HCs), 15 age- and sex-matched, non-smoker subjects without any oral or systemic diseases were included. To study the standards for salivary metabolites, 250 healthy volunteers (103 males, 147 females; mean age 43.2 years, range 21–58 years) were included in the study. The clinical oral examinations of all subjects were carried and cariological status, mucosal alterations, and periodontal status were performed after saliva collection. The demographic data of subjects included in this study is presented in Table 2.

**Table 2:** Demographic data of the study population

Study groups (*N* = number of participants)	Age (years, mean ± SD)	Saliva flow rate ml/min (mean ± SD)	BOP index (mean ± SD)	PI index (mean ± SD)
Patients with OLP(*N* = 15)	37–52 years(45 ± 5.9)	1.41 ± 0.69	0.21 ± 0.23*	0.21 ± 0.23*
Healthy controls (HC) (*N* = 15)	38–52 years(46 ± 5.5)	1.61 ± 0.88	0.09 ± 0.08	0.19 ± 0.17
Standard group^a^ (*N* = 50)	22–58 years(43 ± 6.9)	1.50 ± 1.01	NM	NM

*BOP* bleeding on probing, *PI* plaque index, *NM* not mentioned

*p < 0.05

^a^Pooled saliva

### Saliva collection and sample preparation

Single saliva samples of each participant were collected using standard techniques described previously [38]. Stimulated whole-mouth saliva (SWMS) was collected in the morning between 9 and 10 a.m. to limit circadian variations. All the participants refrained from eating and drinking for at least 1 h before saliva collection. SWMS was collected with masticatory stimulation by chewing neutral paraffin wax (0.9 g; Orion, Espoo, Finland), and then, the patients were asked to collect the drained saliva into a sterile glass cup for 5 min. Saliva flow rates were calculated as mL/min. The collected samples were then centrifuged at 14,000 rpm for 6 min at 4 °C temperature, and the supernatants were stored at − 80 °C for later analysis. Saliva was collected from 250 subjects, were pooled, and used in method validation.

Each saliva sample (450 µl) was mixed with 50 µl of NMR-buffer (1.5 M KH_2_PO_4_, 2 mM NaN_3_, 5.8 mM sodium 3-(trimethylsilyl) propionate-2,2,3,3-d_4_, D_2_O, pH 7.4) and centrifuged at 10,000 × g for 5 min at + 4 °C to delete any solid unneeded parts (debris). The obtained supernatant was then transferred to NMR tubes.

### Data acquisition

Data were acquired using a Bruker AVANCE III HD spectrometer operating at 600.2 MHz (^1^H observation frequency) which was equipped with an inverse selective probe head that includes an automatic tuning and matching unit. The control of the spectrometer occurred by TopSpin 3.2 software (Bruker BioSpin GmbH). The samples are stored at + 6 °C in the sample changer until the measurement. Topshim routine was used to Shimm automatically each saliva sample. After that, samples were preheated to + 25 °C 30 min before the measurement. Every sample is shimmed automatically via Topshim routine. The data were recorded (1D ^1^H data (128 k data points)) at temperature + 25 °C with a 5.8 s repetition time (relaxation time 3.0 s and acquisition time 2.8 s). Before recording, 4 dummy scans using 64 transients with an automatically calibrated 90° pulse to achieve the required signal-to-noise level. A Bruker cpmg1d pulse sequence with t2-filter time of 80 ms and irradiation field of 50 Hz was used to quieten the water peak. For each sample, the automatic calibration of the pulse 90° was used. For all the samples, a constant receiver gain was set.

### Data processing

The acquired spectra were processed and used manual phase correction TopSpin 3.0 software (Bruker BioSpin GmbH). Before Fourier transformations to spectra, the measured free induction decays were multiplied with an exponential window function with a 1.0-Hz line broadening.

A constrained total-line-shape fitting tool in NMR software (PERCH Solutions Ltd, Kuopio, Finland) was used in the metabolite quantification. The PERCH software allows the accurate quantification of identified metabolites even if the signals are overlapping, or the baseline is not linear due to the heavy protein background envelope [39]. An internal reference compound (tri-methylsilyl-propanoic acid, TSP), which had a known concentration was used as an internal standard. For the method validation, pooled saliva samples were used. Obtained final metabolite concentrations are described as µmol/l in saliva.

### Statistics

The Shapiro–Wilk test and the values of kurtosis and skewness were used to test the distribution of metabolic concentrations for normality. Mann–Whitney’s *U* test was used to compare salivary metabolite concentrations between OLP patients and HCs. The metabolites that lacked at least one or more values were not included in the statistical assessment (Table 3). The statistical significance was set at *p* < 0.05. Multivariate discrimination function analysis (DFA) was used to investigate the two metabolites considering together and give maximum discrimination power between the study groups (Discriminant Function Analysis | SPSS Data Analysis Examples (ucla.edu)). All statistical analyses were conducted using SPSS software, version 24.0 (IBM Corp., Armonk, NY, USA).

**Table 3:** Comparison of salivary metabolite concentrations between OLP patients (*N* = 15) and healthy controls. (*N* = 15). The metabolites that lacked at least one value were not included in the statistical assessment

Metabolite	OLP patients mean ± SD	Controls mean ± SD	*p* value	Pooled saliva mean
SCFAs				
Acetate	1971.7 ± 781.1	1503.0 ± 715.2	0.048*	1687.0
Butyrate	23.2 ± 20.9	16.4 ± 10.9	0.314	15.5
Formate	152.0 ± 195.0	80.1 ± 63.3	0.192	168.0
Propionate	284.3 ± 205.7	235.7 ± 156.2	0.472	248.0
Amino acids				
Alanine	13.8 ± 13.2	11.4 ± 7.3	0.267	14.3
Glycine	50.9 ± 25.5	51.5 ± 35.4	0.871	82.8
Phenylalanine	9.3 ± 5.4	8.5 ± 3.6	0.818	11.5
Taurine	110.5 ± 49.6	48.3 ± 29.0	0.271	38.7
Proline	133.5 ± 114.5	59.1 ± 44.1	0.212	116.1
Histidine	23.3 ± 11.8	-	-	-
Tyrosine	24.6 ± 12.5	89.6 ± 11.4	0.003**	103.3
Organic acids				
Citrate	19.1 ± 7.9	18.3 ± 13.8	0.996	22.2
Lactate	265.6 ± 140.4	198.3 ± 174.5	0.255	155.3
Pyruvate	17.6 ± 11.2	10.4 ± 7.8	0.032*	12.8
Succinate	33.8 ± 32.1	28.2 ± 15.7	0.549	18.9
Amines				
Methylamine	12.1 ± 1.3	2.0 ± 0.9	0.006**	1.5
Trimethylamine	1.4 ± 1.4	-	-	-
Other				
Choline	9.4 ± 5.3	4.1 ± 2.0	0.292	4.2
Isopropanol	2.47 ± 0.9	-	-	-
1.2-propanediol	-	27.3 ± 14.2	-	19.1
Butanol	17.1 ± 8.1	8.6 ± 5.8	0.126	15.5
Methanol	28.5 ± 17.5	27.7 ± 9.2	0.878	22.6
Fucose	46.5 ± 30.9	66.3 ± 40.3	0.167	62.8

*SCFAs* short chain fatty acids

**p < 0.05

## Result

The present study included the saliva samples of 15 OLP patients and 15 age- and sex-matched HCs. Stimulated whole saliva flow rates did not differ significantly between the study groups. The indexes of periodontal health (bleeding on probing, BOP; plaque index, PI) are higher in patients with OLP. The demographic data of subjects included in this study are presented in Table 2.

A total of 23 metabolites and their concentrations were detected in saliva samples as presented in Table 3. In the statistical analysis, the median concentrations of acetate, pyruvate, and methylamine were significantly higher (*p* = 0.048, *p* = 0.032, *p* = 0.006, respectively) in OLP patients compared with HCs. In contrast, the tyrosine concentration was significantly lower (*p* = 0.003) in the saliva samples of OLP patients. Other metabolites found in the study result did not show any statistically significant differences between OLP and controls.

**Fig. 2:**
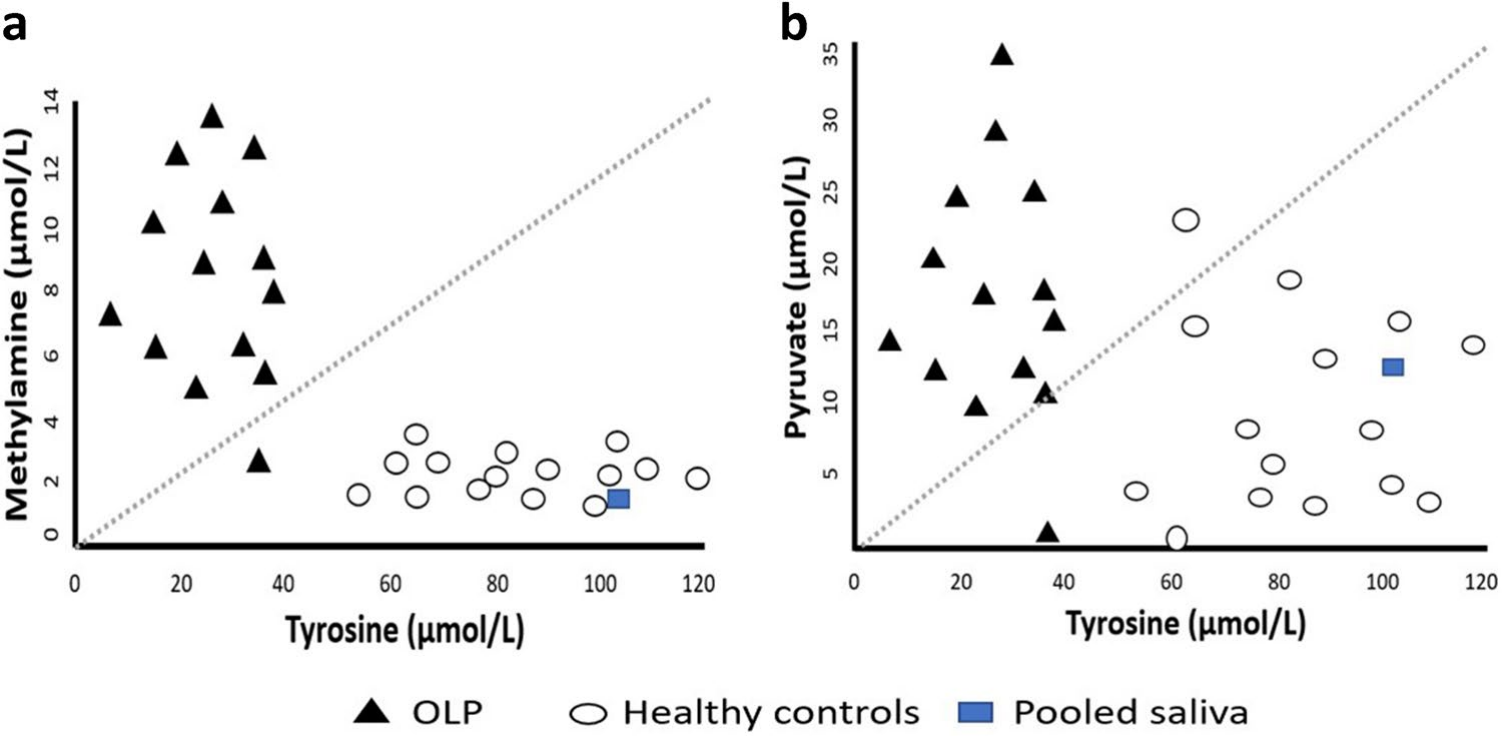
Scatter plot (**a**, **b**) presenting the salivary tyrosine concentration against salivary methylamine and pyruvate concentrations for 14 OLP patients and 15 control subjects

Metabolite comparisons in the DFA analysis, a pair of methylamine and tyrosine resulted in the highest discriminant power (96.7%) (Fig. 2). The combination of pyruvate and tyrosine provided also strong discriminant power (93.5%). One saliva sample of an OLP patient was removed from the analysis because it contained significantly high concentrations of methylamine and pyruvate distorting the result.

## Discussion

This preliminary study is conducted to identify the potential salivary biomarkers of OLP using NMR spectroscopy. In this study, OLP patients with a lower accumulation of inflammatory cells (mild degree inflammation) were selected while patients with clinical ulceration and erosive changes were not enrolled in the study. In addition, patients in the study had no medications for oral symptoms. When compared with periodontal indexes, these were higher in OLP patients than in healthy. This indicated a mild gingivitis or periodontitis without marked clinical symptoms in OLP patients. We present several metabolites showing the potential to differentiate OLP from controls based on NMR spectroscopy analysis, which makes an important clinical implication. NMR is one of the analytical techniques used in metabolomics due to its high reproducibility and unbiased low-molecular-weight salivary quantification [40]. The univariate analysis showed the up-regulation of three metabolites: acetate, methylamine, and pyruvate, whereas tyrosine was down-regulated in patients with OLP. In the multivariant analysis, a significant combination of tyrosine together with methylamine provided maximum discrimination among OLP patients and HCs.

Acetate is the most common building block for biosynthesis, such as fatty acids. An increased level of acetate indicates the infiltration of bacterial species that are capable of proteolytic destruction and producing short-chain fatty acids (SCFAs). Acetate, propionate, n-butyrate, and succinate are some of SCFAs, which are immunomodulators. The previous study reported the increase in SCFAs in the saliva of chronic periodontitis patients and strongly correlated it with the disease severity, gingival inflammation, and the total microbial load [41, 42]. Some oral bacterial species have shown that lactate can metabolize to acetate and propionate hence the level increases in saliva [43]. Acetate, propionate, and butyrate are recognized as the products of bacterial fermentation in the gut and have multiple important roles in general health [44]. The increase in the acetate profiles in OLP patients may reflect the change in the functional activity of microbes and their interaction with host oral tissue.

In the present study, OLP patients had shown an increase in bleeding on probing and plaque index compared to control patients. The observed periodontal status in the OLP patients confirms the SCFA production by the periodontal pathogens and the release of metabolites from the infection site into the oral microenvironment. As reported earlier, the inflammation caused by the periodontal disease is not limited to the periodontal tissues. Instead, it encompasses other immunological and inflammatory processes to produce inflammatory mediators such as cytokines, interleukins, prostaglandins, and c-reactive protein. The released mediators can spread through bloodstreams and can cause systemic inflammation [45]. Rheumatoid arthritis, an autoimmune inflammatory disease, is correlated with the periodontal status, though the exact mechanism remains unknown [46]. Several studies have presented autoimmune and inflammatory-mediated destruction as an important pathogenesis of OLP and periodontal disease. However, there is a lack of consensus between their interrelation [47, 48]. With our result, we could observe that the periodontal status was deteriorated in OLP patients, and the periodontal pathogens were another source for the salivary metabolites. On the other hand, it is difficult to comment on the direct relationship between OLP and periodontal disease or vice-versa.

Methanogens are anaerobic prokaryotic microorganisms responsible for hydrogenotrophic metabolism. It utilizes hydrogen to reduce carbon dioxide to methane, a process called methanogenesis [49]. Methylamine is a substrate for methanogenesis. A study report from the human gut showed that the methanogenesis substrates are mostly obtained from bacterial fermentation [50]. Later, the presence of higher levels of methanogens in the oral cavity of periodontitis subjects was reported [51]. Apart from methanogens, the choline metabolism by gut bacteria exposes humans to potentially harmful methylamines. Choline can lead to an increased level of methylamines, which could be a substrate for the formation of nitrosamines, known to have carcinogenic activity [52]. Amines, like methylamine, dimethylamine, and trimethylamine, are shown to readily convert into toxic metabolites like *N*-nitroso-dimethylamine in the presence of sodium nitrite obtained from food products [53]. The metabolites analyzed in our study did not present any change in the choline and trimethylamine. The high level of salivary methylamines in OLP patients may be caused by combined effects of diet and the end products of oral microbial metabolism.

Pyruvate is commonly encountered as one of the end products of glycolysis. It is transported to mitochondria to participate in the citric acid cycle and for ATP generation to augment several biosynthetic pathways [54]. The increased glycolysis produces more of its end products such as pyruvic acid and lactic acid. As lactic acid is unstable, it converts quickly back to pyruvate, hence, leading to an overall increase in the expression of pyruvate. Such excess pyruvate escapes into the blood or the saliva [55]. In hypoxic situations, pyruvate can undergo fermentation to produce lactate. Both pyruvate and lactate can be used to regenerate glucose and maintain cellular homeostasis. Pyruvate can also be involved in the anabolic synthesis of fatty acids and amino acids. There is also growing evidence that it can directly influence nuclear activity and epigenetic modifications, forming the interface between the genome and the metabolic state of the cell [56, 57]. Pyruvate increase in the saliva of OLP could be due to (1) the presence of oral microorganisms using only the glycolysis pathway for their energy and survival or (2) more release of pyruvate in saliva. Overall, the cellular micro-environment is affected by the altered pyruvate level in OLP.

Salivary tyrosine belongs to a group of aromatic amino acids and is one of the promising biomarkers for oxidative stress [58]. The reduced level of tyrosine is observed in OLP patients which might have imbalanced the oxidative and anti-oxidative status. Tyrosine is believed to participate in the activity of intracellular signaling pathways mediated by tyrosine kinases or transcription factors. Hence, it maintains the normal functions of proteins involved in the preservation of redox balance in cells [59, 60]. Tyrosine was considered a biochemical marker of inflammation in OLP where a link between tyrosine modification and immunoreactive was established. Also, it is believed that tyrosine can participate in PI3K/Akt signaling, NF-kB and p53 transcription factor activation, that forms a basis for malignant transformation [60, 61]. Tyrosine modifications in OLP can intensify oxidative stress that can disturb the structure and physiological function of numerous proteins. The reduced salivary tyrosine levels might cause a negative effect on the antioxidant properties in OLP. The detection of salivary tyrosine could be a promising marker in OLP.

The presence of endogenous and exogenous proteases in salivary glands, exfoliating cells, and oral microflora can produce free salivary amino acids [62]. Proteolytic bacteria can break down proteins and peptides into amino acids and convert them into SCFAs which contributes to the change in salivary organic acid content [63]. Thus, the observation of our result reflects the bacterial metabolic pathways that caused the altered concentration of salivary amino acids and organic acids in OLP patients. It should be noted that the oral cavity is a critical place for oxidative stress generated by both exogenous and endogenous sources. The exogenous sources include oral tissue exposure to thermal, chemical, and microbial stimuli, and the endogenous sources refer to chronic or acute infections in oral tissue, such as periodontitis and OLP [64]. Oxidative and nitrosative stresses have been shown to play a significant role in the pathogenesis of OLP and its malignant potential. The inflammatory cells in OLP are known to produce reactive oxygen species (ROS), which in turn intensifies the inflammatory response [65]. Therefore, noninvasive monitoring of the potential salivary biomarkers of OLP could play a key role in early detection, prognosis, treatment planning, and monitoring the treatment success.

## Conclusion

The present study provides a preliminary report on salivary metabolites in patients with OLP. The advantage of this study is the accurate patient selection. The age and sex-matched controls were included in the study. All patients included in the study were without any medications and any general diseases. Also, patients had no clinical symptoms or any other oral symptoms. Hence, factors that could affect the salivary metabolite profile were minimized except for mild gum disease. The disadvantage of this study is the limited number of subjects studied. In the future, analyses on salivary metabolites and host response in OLP patients are necessary to improve our understanding about the pathways in the oral cavity and the disease progression.

## Data Availability

The datasets used and analyzed during the current study are available from the corresponding author upon reasonable request.
